# Joint-tissue integrative analysis identifies high-risk genes for Parkinson’s disease

**DOI:** 10.3389/fnins.2024.1309684

**Published:** 2024-03-21

**Authors:** Ya-Shi Wu, Wen-Han Zheng, Tai-Hang Liu, Yan Sun, Yu-Ting Xu, Li-Zhen Shao, Qin-Yu Cai, Ya Qin Tang

**Affiliations:** ^1^Department of Bioinformatics, School of Basic Medical Sciences, Chongqing Medical University, Chongqing, China; ^2^Department of Cell Biology and Medical Genetics, School of Basic Medical Sciences, Chongqing Medical University, Chongqing, China

**Keywords:** Parkinson’s disease, MR-JTI, GWAS, TWAS, Mendelian Randomization

## Abstract

The loss of dopaminergic neurons in the substantia nigra and the abnormal accumulation of synuclein proteins and neurotransmitters in Lewy bodies constitute the primary symptoms of Parkinson’s disease (PD). Besides environmental factors, scholars are in the early stages of comprehending the genetic factors involved in the pathogenic mechanism of PD. Although genome-wide association studies (GWAS) have unveiled numerous genetic variants associated with PD, precisely pinpointing the causal variants remains challenging due to strong linkage disequilibrium (LD) among them. Addressing this issue, expression quantitative trait locus (eQTL) cohorts were employed in a transcriptome-wide association study (TWAS) to infer the genetic correlation between gene expression and a particular trait. Utilizing the TWAS theory alongside the enhanced Joint-Tissue Imputation (JTI) technique and Mendelian Randomization (MR) framework (MR-JTI), we identified a total of 159 PD-associated genes by amalgamating LD score, GTEx eQTL data, and GWAS summary statistic data from a substantial cohort. Subsequently, Fisher’s exact test was conducted on these PD-associated genes using 5,152 differentially expressed genes sourced from 12 PD-related datasets. Ultimately, 29 highly credible PD-associated genes, including CTX1B, SCNA, and ARSA, were uncovered. Furthermore, GO and KEGG enrichment analyses indicated that these genes primarily function in tissue synthesis, regulation of neuron projection development, vesicle organization and transportation, and lysosomal impact. The potential PD-associated genes identified in this study not only offer fresh insights into the disease’s pathophysiology but also suggest potential biomarkers for early disease detection.

## 1 Introduction

Parkinson’s disease (PD), the second most prevalent progressive neurodegenerative disorder globally, affects over 6 million individuals worldwide, and its prevalence continues to escalate rapidly. Projections indicate that it might even double within the next 30 years (GBD 2016 Neurology Collaborators, 2019). PD is recognized as a movement disorder characterized by symptoms such as rigidity, slowness, and tremor (Armstrong and Okun, 2020). Most PD cases manifest in individuals aged between 85 and 89, with men being more susceptible than women (Tolosa et al., 2021). The main causes of PD are understood to be a combination of environmental and genetic factors. Instances of severe brain injury, consumption of dairy products, and exposure to pesticides have all been associated with an increased risk of PD (Ascherio and Schwarzschild, 2016). Studies suggest that the complex interaction between environmental and hereditary factors affecting essential cellular processes is the major cause of PD (Kalia and Lang, 2015).

Through extensive cohort studies on PD employing Genome-wide Association Studies (GWAS), a robust technique for detecting complex diseases, researchers have identified over 70 common susceptibility genes associated with PD (Nalls et al., 2019). As early as 2009, a comprehensive genetic risk survey of Parkinson’s disease, using European population GWAS data, revealed numerous additional risk sites and provided essential insights into the pathogenesis of PD (Simón-Sánchez et al., 2009). While these findings have successfully linked numerous genetic loci to various complex features in PD, providing a significant framework for PD research, the presence of strong linkage disequilibrium (LD) often obscures the causal relationships between genes and phenotypes, posing challenges in interpreting GWAS statistics alone (Gallagher and Chen-Plotkin, 2018). To address this issue, many researchers have turned to the Transcriptome-wide Association Study (TWAS) approach, a valuable development that combines individual gene expression data with GWAS results. This allows for the quantitative prediction of gene expression levels in specific diseases, aiding the identification of genes with potential biological functions and enhancing the understanding of the relationship between genes and PD.

The traditional TWAS approach generally involves three steps (Gamazon et al., 2015; Gusev et al., 2016; Barbeira et al., 2018). (A) Model training, A model is fitted for the expression quantitative trait of the target gene based on reference data (e.g., GTEx database or other cohort data), with nearby genotypes such as Single Nucleotide Polymorphisms (SNPs) as predictive variables. (B) Gene expression filling, employing the training model to fill in missing gene expression data within large-scale GWAS cohorts. (C) Correlation analysis, the gene expression after filling is used to analyze the association between genes and disease traits (Luningham et al., 2020). However, researchers are continually advancing beyond the traditional TWAS approach to identify genes associated with complex pathological features that exhibit robust associations. Enhanced detection techniques include TIGAR (Nagpal et al., 2019), PrediXcan (Gamazon et al., 2015), PMR-Egger (Yuan et al., 2020), SMR/HEIDI (Pavlides et al., 2016; Zhu et al., 2016; Hauberg et al., 2017), Sherlock (He et al., 2013), eCaviar (Hormozdiari et al., 2016), enloc (Wen et al., 2017), and RTC (Nica et al., 2010). In recent years, there has been an increasing trend in employing traditional or improved TWAS approaches to identify candidate causal genes for PD. For instance, Yao et al. (2021) integrated GWAS results with the eQTL data of 13 brain tissues for TWAS analysis to identify significant associated genes for PD. Similarly, Li et al. (2019) conducted TWAS analysis based on RNA splicing or splicing QTL (sQTL) to uncover additional connections between genes and PD.

**Figure 1:**
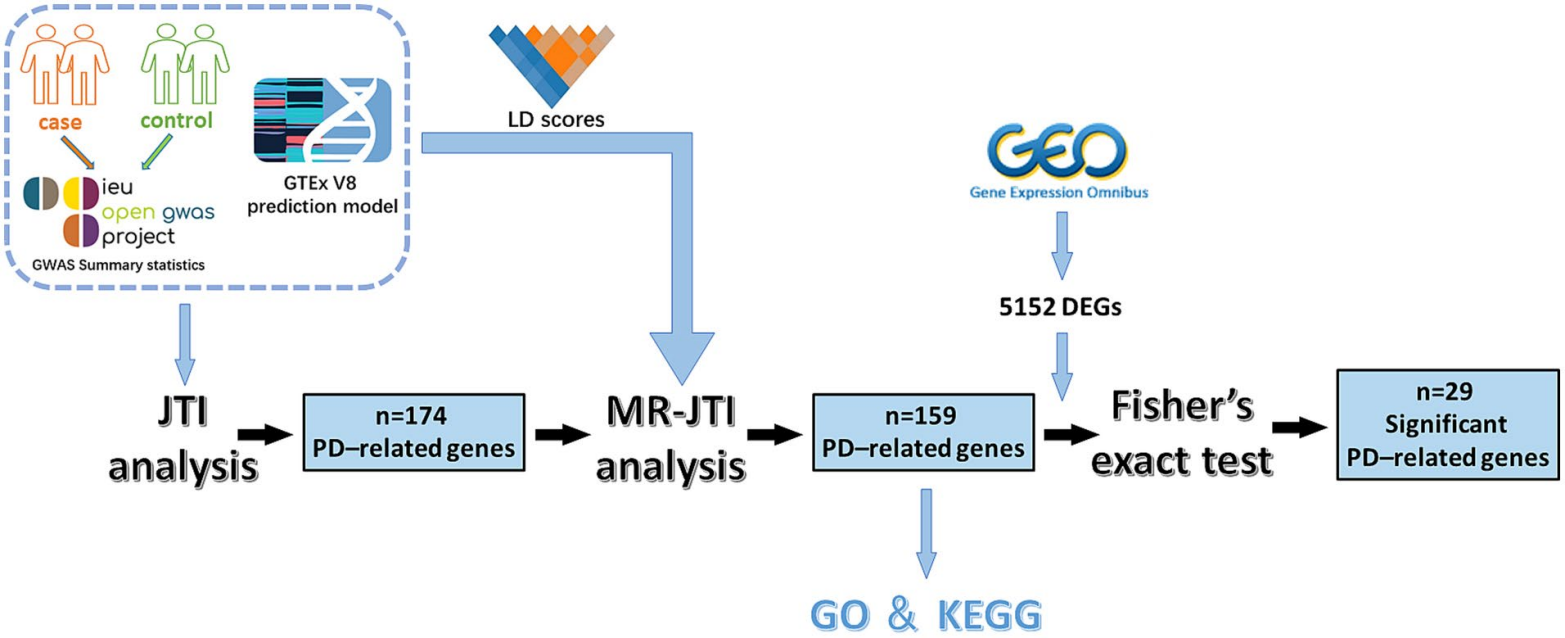
The experimental flow chart of this study. This study involved several key steps. Initially, GWAS data and JTI pre-training models were integrated for TWAS analysis. This process yielded 174 PD-related genes. Subsequently, through further analysis combining LD score, GTEx V8 eQTL data, and GWAS summary statistics, the MR approach identified 159 PD-associated risk factors. To enhance reliability, Fisher’s exact test was conducted on 5,152 DEGs (*p* < 0.05 and |log_2_FC| > 1) obtained from the GEO dataset of PD and 159 PD-associated risk factors, resulting in the identification of 29 significant PD-related risk genes.

In 2020, Zhou and colleagues introduced MR-JTI as an enhanced version of the PrediXcan TWAS method. This innovative approach amalgamates the Joint-Tissue Imputation (JTI) technique with the Mendelian Randomization (MR) framework for causal inference (Zhou et al., 2020). Unlike conventional TWAS methods, JTI harnesses data from transcriptomes of various tissues (such as the GTEx V8 panel) and shared regulatory gene maps to elucidate the structure of the expressed genome and ascertain the association between expression and traits. For JTI, the prediction model was generated using a reference multi-tissue transcriptome panel, and the predictive performance was evaluated in each target tissue through cross-validation. When the transcriptional regulation of target genes exhibits specificity in simple tissues, it will automatically revert the model to a single-tissue PrediXcan prediction model. JTI significantly enhances prediction performance in a tissue-specific manner, surpassing traditional PrediXcan and UTMOST (multi-organization interpolation method) methodologies (Zhou et al., 2013). In trait mapping applications, prediction models can be applied to GWAS summary statistics to identify robust gene-level associations. In this study, we leveraged the JTI scheme, GWAS data and eQTL cohorts from GTEx (version 8) were utilized to perform tissue-specific TWAS analysis for PD-related genes in 13 brain regions, and the results were incorporated into the MR framework to identify the causal relationship between risk factors and PD to enhance the reliability of obtained results. The experimental flow diagram is shown in Figure 1. Our study will contribute to identifying more precise potential therapeutic targets and biomarkers for PD.

## 2 Materials and methods

### 2.1 GWAS data for PD

The GWAS data for PD involving 33,674 patients and 449,056 control subjects were obtained from the study conducted by Nalls et al. (2019). The data were downloaded from the European Bioinformatics Institute GWAS Catalog^1^ with GWAS ID number ieu-b-7. The data are accessible via https://gwas.mrcieu.ac.uk/. The comprehensive details regarding sample collection and analysis methods can be found in the original article published in The Lancet Neurology (Nalls et al., 2019).

### 2.2 TWAS analysis of PD using the JTI method

The GTEx database serves as a substantial repository of human genetic information and is continually updated to offer the latest insights. Its most recent iteration, GTEx V8, contains comprehensive sequencing data from 54 non-diseased tissue sites collected from 948 donors (available for download from the GTEx project website)^2^ (Battle et al., 2017). Through a comprehensive assessment of the shared regulatory architecture of gene expression across different tissues and the unique genetic regulation in specific tissue, the JTI approach developed by Zhou et al. (2020) significantly enhances prediction performance while effectively controlling type I error rates. The pre-training JTI prediction models (eQTL models) for each tissue based on the JTI scheme and GTEx V8 data, alongside related eQTL summary statistics and SNP-SNP covariance matrices, all accessible via https://zenodo.org/record/3842289#.Y9. A total of 13 brain region-specific pre-trained prediction models were obtained, encompassing areas such as the brain amygdala, anterior brain, caudate brain, cerebellar brain, cerebellum, cortex, frontal brain, hippocampus, hypothalamus, brain nucleus, putamen brain, spinal brain, and substantia brain.

Subsequently, utilizing the JTI method, we employed the pre-trained model and GWAS data to conduct TWAS analysis, aiming to identify PD-related risk genes. All *p*-values for PD-related risk genes obtained through JTI underwent adjustment using the Bonferroni method via the p.adjust function in R (version 4.1.3) and the false discovery rate (FDR) method (genes with FDR < 0.05 were defined as being associated with PD).

### 2.3 JTI analysis method combined with MR

Though the relationship between gene expression and PD has been established by JTI, it is still uncertain whether the differential expression of these genes is the cause or consequence of PD. Therefore, the JTI results were then incorporated into the MR framework to evaluate the causal relationship between gene expression and PD. Initially, tackling the challenge of LD bias, we addressed this issue by computing LD scores, using GCTA software. These LD scores were generated by imputing data from the 1,000 Genomes Project into GCTA. The LD score matrices, which gauge the degree of association between loci based on allele frequency and correlation coefficients, were calculated using publicly available samples from individuals self-identified as healthy.^3^ GCTA64 (Yang et al., 2011) was employed to compute LD scores from the GTEx genotype data. Subsequently, the LD scores, effect size of eQTL (beta), standard error (SE) of eQTL effect size, eQTL *p*-value and GWAS beta, and GWAS *p*-value were then used as the input of MR. The primary outcome of MR-JTI was the “expression” significance, denoting the significance (*p*-value <0.05) of the causal relationship between gene expression and PD. Ultimately, the MR-JTI method was instrumental in identifying potential PD-related causal genes.

### 2.4 Fisher’s exact test for potential risk factors of PD

The Gene Expression Omnibus (GEO), an openly available genomic data repository^4^, offers a vast collection of genetic data, encompassing complete gene expression profiles, chips, and microarrays. In this study, we utilized the GEO database to retrieve and download the 12 publicly accessible datasets related to PD that were used (Table 1). According to the high consistency in gene expression patterns among these brain regions, we integrated 12 GEO datasets, which provide a more comprehensive data foundation to screen PD risk factors identified by MR-JTI analysis conducted using Fisher’s exact test. Differentially expressed genes (DEGs) were screened based on *p* < 0.05 and |log_2_ fold-change (FC)| > 1. Annotation of the gene symbols in the datasets was performed using DAVID online software^5^. The potential risk factors of PD identified by MR-JTI and the DEGs related to PD were analyzed using Fisher’s exact test to further refine the selection of these risk factors.

**Table 1:** Details of PD-related datasets.

GEO accession	Public date	Tissues	Control	PD case	References
GSE205450	May, 2023	Caudate and putamen regions of the striatum	80	70	Irmady et al. (2023)
GSE8397	Jan, 2008	Substantia nigra/Superior frontal gyrus	18	29	Duke et al. (2007)
GSE168496	Jan, 2023	Substantia nigra	8	8	Tranchevent et al. (2023)
GSE106608	May, 2021	Subthalamic nucleus	9	7	Not published yet
GSE163176	June, 2021	Brain slice	3	3	Lian et al. (2021)
GSE136666	Sep, 2020	Putamen/Substantia nigra	8	8	Xicoy et al. (2020)
GSE133101	June, 2020	Amygdala/Mediltemporal Gyrus/Substantia nigra	26	43	Hanan et al. (2020)
GSE134390	Feb, 2020	Putamen	0	20	Not published yet
GSE114517	Apr, 2020	Substantia Nigra/Amygdala/Mediltemporal Gyrus	29	46	Simchovitz et al. (2020)
GSE42966	Sep, 2021	Substantia nigra	6	9	Quan et al. (2021)
GSE28894	Sep, 2021	Frontal Cortex/Cerebellum/Medulla	59	55	Chis et al. (2021)
GSE7621	June, 2007	Substantia nigra	9	16	Lesnick et al. (2007)

### 2.5 Functional enrichment analysis of PD risk genes

Gene Ontology (GO) enrichment analysis, including biological pathways (biological process, BP), cellular components (CC), and molecular function (MF), and the Kyoto Encyclopedia of Genes and Genomes (KEGG)^6^ were employed to conduct functional enrichment analysis of the genes associated with PD. These analyses were performed using the R package ClusterProfiler. The significantly enriched pathways (adjusted *p* < 0.05) were visualized using the R package Circlize.

## 3 Results

### 3.1 MR-JTI revealed 159 PD-related genes

Studies estimating PD heritability through twin and family analyses suggest a significant role for genetic factors in driving phenotypic variance, ranging between 27 and 60% (Do et al., 2011). To determine the risk loci connected to PD, utilizing GWAS statistic data for PD from Nalls et al., we conducted the JTI analysis using eQTL pre-training models to uncover risk genes linked to PD. After the elimination of duplicate genes, 174 candidate genes associated with PD risk (FDR < 0.05, Supplementary Table 1) were identified from 13 different brain regions, suggesting that their expression might be tied to the genetic risk of developing PD.

**Figure 2:**
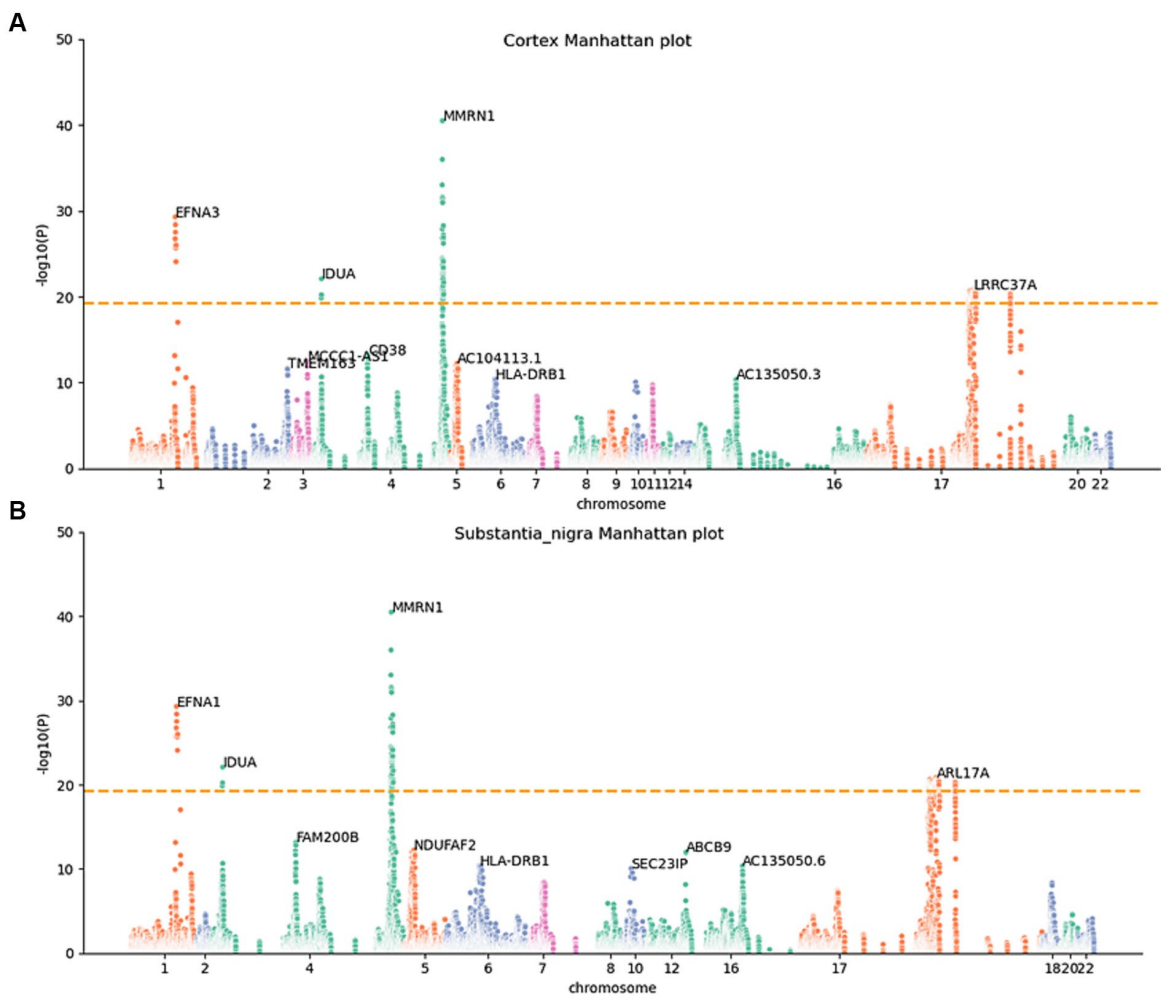
Manhattan maps from MR-JTI results of different brain regions. The Manhattan maps derived from MR-JTI results illustrated the relationship strength between genes and PD in different brain regions. The “-log (*p*-value)” of each gene in the JTI result is represented on the vertical axis; the higher values indicated a stronger association between the gene and PD. Brain cortex **(A)** and substantia nigra **(B)**. The dashed lines on each graph represent a significance cutoff threshold of 5e-20.

To determine whether the differential expression of these genes associated with PD is a cause or a consequence of PD development, we utilized the MR analysis approach. This involved integrating LD scores, eQTL data, and GWAS summary statistics into the MR analysis, aiming to evaluate the genes identified using the JTI method. Subsequently, 159 probable causative risk genes for PD (FDR < 0.05, Supplementary Table 2) were pinpointed across the 13 brain areas after the removal of duplicate genes. The Manhattan plot represents the risk genes of PD screened by MR-JTI from 13 brain regions (Figure 2 and Supplementary Figure 1). Among PD-related genes in the brain substantia area, ARSA (Senkevich et al., 2023), KANSL1-AS1 (Lona-Durazo et al., 2023), FAM47E (Blauwendraat et al., 2019), and ARHGAP27 (Saeed, 2018) have been reported to be risk loci that contribute to the development of PD. The genetic alteration among NMRN1 (Fuchs et al., 2007), CRHR1 (Cheng et al., 2020; Rasmi et al., 2023), and HLA-DRB1 (Le Guen et al., 2023) in the brain cortex increased the risk of PD.

### 3.2 Gene enrichment for PD

**Figure 3:**
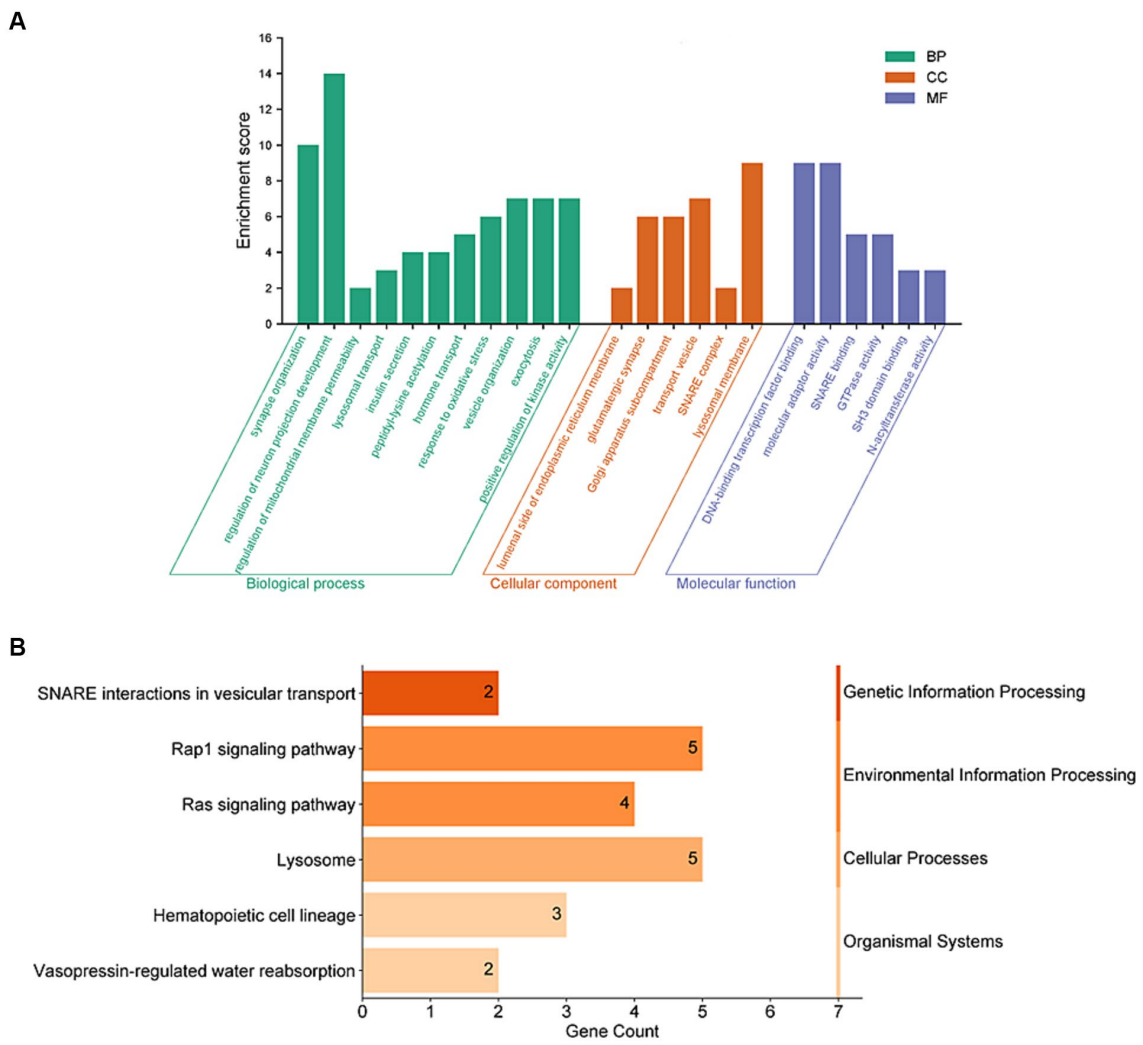
Functional enrichment analyses of PD-related risk factors. **(A)** GO enrichment analysis of the 159 PD-related genes, including molecular biological process (BP), cellular component (CC), and function (MF). **(B)** Analysis of KEGG enrichment associated with the 159 PD-related genes.

To further understand the connection between these 159 genes and PD, we conducted enrichment analyses using GO and KEGG functional enrichment analysis. The results of GO enrichment analysis revealed significant enrichment in synaptic tissue pathways (such as synaptic vesicle exocytosis and endocytosis and presynaptic and postsynaptic regions), protein acetylation, kinase activity regulation, and lysosomal function. Notably, pathways related to neuronal projection development and vesicular tissue transport involving genes like CD38, EFNA1, STX1B, and SNCA were highlighted (Figure 3A and Supplementary Table 3). Furthermore, the KEGG functional enrichment analysis unveiled enrichments in lysosome, vesicular transport, signaling pathways, and hematopoietic cell lineage among the 159 genes associated with PD (Figure 3B and Supplementary Table 4).

### 3.3 Fisher’s exact test for DEGs and risk genes linked with PD

**Figure 4:**
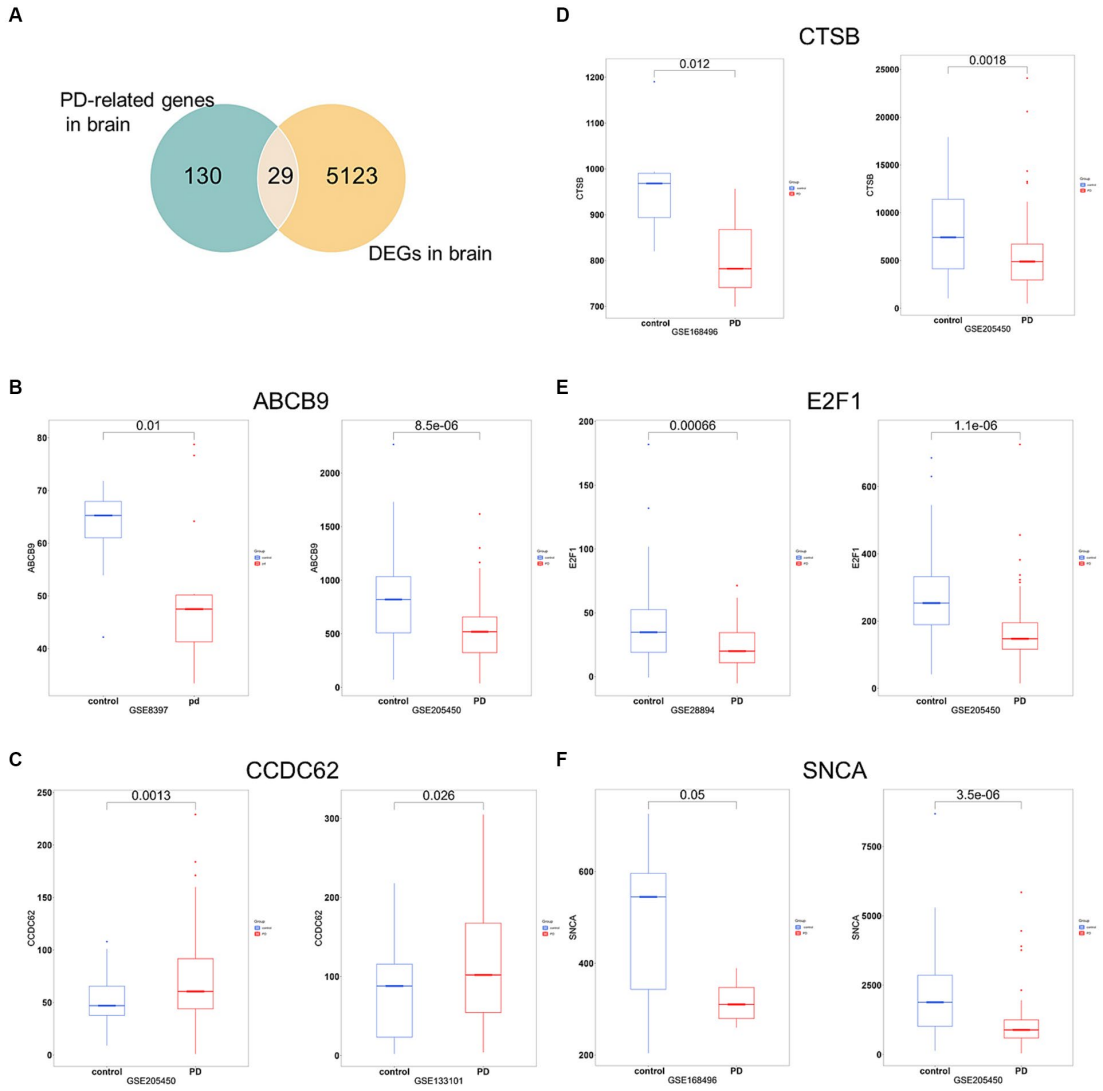
Significant genes linked with PD. **(A)** Venn analysis highlights 29 significant genes shared between the pool of 159 PD-related genes and the 5,152 DEGs obtained from brain regions. **(B–F)** Boxplots displayed the expression trends of ABCB, CCDC62, CTSB, E2F1, and SNCA in two datasets (*p* < 0.05). PD, PD cases; control, normal control cases.

To increase the accuracy of identifying risk factors for PD, we performed Fisher’s exact test on 159 PD-associated genes and the integrated DEG datasets in PD. Performing DEG analysis on 12 PD-related datasets sourced from distinct brain regions obtained from the GEO database (refer to Table 1), we identified a total of 5,152 distinct DEGs meeting the criteria (*p* < 0.05 and |log_2_FC| > 1, Supplementary Table 5). Using Venn analysis to compare the 159 PD-related genes with the 5,152 DEGs found in different brain regions, we identified 29 significant genes associated with PD (Figure 4A). We evaluated the correlation between the 29 genes and PD by manually retrieving published literature, providing suggestions for future research on the role of these significant genes in PD. The functions of these 29 genes involved in PD pathophysiology are detailed in Table 2. Moreover, from the pool of 29 genes, we randomly selected ABCB9, CCDC62, CTSB, E2F1, and SNCA to serve as representative genes, illustrating their expression trends in PD cases and normal control subjects (Figures 4B–F).

**Table 2:** The specifics of the significant genes of PD.

Gene symbol	Full name	Function in PD	References
ABCB9	ATP binding Cassette Subfamily B member 9	ABCB9 methylation associated with PD	Chuang et al. (2017)
AK7*	Adenylate Kinase 7	N/A	Not published yet
ARHGAP27*	Rho GTPase Activating Protein 27	N/A	Saeed (2018)
ARL17A*	ADP Ribosylation Factor like GTPase 17A	N/A	Not published yet
ARL17B	ADP Ribosylation Factor like GTPase 17B	ARL17B was associated with negative control of neuron projection development.	Tian et al. (2023)
ARSA	Arylsulfatase A	ARSA variations may be linked to PD and serve as a molecular chaperone for SNCA.	Senkevich et al. (2023) and Lee et al. (2019)
CCDC62	Coiled-coil Domain Containing 62	CCDC62 gene polymorphisms have a statistically significant connection with PD. Using psychotropic drugs may decrease PD risk through CCDC62 transcription.	Yi et al. (2023) and Lauterbach (2012)
CFAP119*	Cilia and Flagella Associated Protein 119	N/A	Not published yet
CRHR1	Corticotropin Releasing Hormone Receptor 1	CRHR1 is a recognized therapeutic target and its methylation may introduce potential pathophysiology of PD.	Cheng et al. (2020) and Rasmi et al. (2023)
CTSB	Cathepsin B	CTSB is involved in lysosomal autophagy, which demonstrates that cellular clearance system malfunction plays a role in the etiology of PD.	Bellomo et al. (2020) and Senkevich and Gan-Or (2020)
E2F1	E2F Transcription Factor 1	An increase in the expression of E2F-1 after the cell cycle has been initiated may cause neuronal apoptosis, which is a characteristic of PD.	Folch et al. (2012) and Verdaguer et al. (2007)
EFNA3	Ephrin A3	EFNA3 took part in neurodevelopment. Appropriate dopaminergic (DA) neuron development from transplanted cells and accurate axon growth are two fundamental concepts behind effective cellular treatments for PD. Varying expression levels of EFNA3, which direct axon growth and aid in DA neuron differentiation, offer a novel concept for the therapy of PD.	Tirozzi et al. (2023) and Wang et al. (2016)
FAM47E	family with sequence similarity 47 member E	FAM47E was a known PD risk locus, exhibited a significant effect after Bonferroni correction.	Blauwendraat et al. (2019)
FBXO34*	F-box protein 34	N/A	Not published yet
FMNL1	Formin like 1	FMNL1 was identified as a biomarker linked to PD	Hu et al. (2022)
HLA-DRB1	Major Histocompatibility Complex, Class II, DR beta 1	Spontaneous PD has been linked to polymorphisms in the HLA-DR region. By potentially working against tau, an adaptive immune response mediated by HLA-DRB1 lowers the risk of PD and AD and opens up prospective treatment options.	Le Guen et al. (2023)
HOPX*	HOP Homeobox	N/A	Not published yet
ITGA3*	Integrin Subunit alpha 3	N/A	Not published yet
KANSL1-AS1	KANSL1 Antisense RNA 1	KANSL1-AS1 has a negative correlation with adaptive immune cells in PD.	Lona-Durazo et al. (2023)
LAT	Linker for Activation of T cells	Carrier of SGK1, which aids in the development of PD.	Lang et al. (2010)
LINC01102*	Long Intergenic Non-protein Coding RNA 1102	N/A	Not published yet
MMRN1	Multimerin 1	MMRN1 causes early-onset PD.	Fuchs et al. (2007)
NSF	N-ethylmaleimide Sensitive Factor	NSF protein aggregation is a characteristic of PD.	Pischedda et al. (2021)
PDLIM2*	PDZ and LIM domain 2	N/A	Not published yet
PRSS36	Serine Protease 36	PRSS36 was reported as a risk factor for PD.	Dang et al. (2022)
SEC23IP	SEC23 interacting protein	SEC23IP was reported as a risk factor for PD.	Gaare et al. (2020)
SNCA	Synuclein alpha	SNCA expression is the main contributor to neurotoxicity and protein aggregation, which are neuropathological hallmarks of PD.	Du et al. (2020), Ferese et al. (2015), and Dehay et al. (2015)
SPPL2C	Signal Peptide Peptidase Like 2C	SPPL2C variations in the MAPT gene raise a fresh hypothesis for further research into PD.	Soto-Beasley et al. (2020)
STX1B	Syntaxin 1B	STX1B polymorphisms are associated with PD.	Wang et al. (2015)

*New potential PD-related gene has not been reported as a function of PD.

## 4 Discussion

Given the complex etiology of PD and the lack of effective drug targets, there is a lack of effective treatment options and intervention strategies in clinical practice. In this study, we conducted TWAS utilizing JTI to uncover potential PD risk factors. Across 13 distinct brain areas, this analysis identified a total of 174 potential genes associated with PD risk. Subsequent causal inference using MR revealed 159 genes strongly linked to PD. The prevailing theory regarding PD pathophysiology underscores the depletion of dopaminergic neurons in the substantia nigra and the accumulation of α-synuclein and other neurotransmitters in the Lewy body as key factors (Hirtz et al., 2007; Kalia and Lang, 2015). Interestingly, among the 159 identified risk genes for PD, several are enriched in these PD-related pathways. For example, genes such as SCNA, CDC42 (Ying et al., 2022), EFNA1, MAPT (Aarsland et al., 2017), and LZTS3 (Li et al., 2023). Specifically, overexpressed SCNA, which codes for the protein alpha-synuclein, displayed aberrant synaptic nucleoprotein aggregation, causing neurotoxicity and neuronal death in PD (Rocha et al., 2018). EFNA1 has been observed to influence dopaminergic neurogenesis and angiogenesis in PD rat models, potentially affecting PD risk (Jing et al., 2012). It was worth noting that many genes were enriched in mitochondrial-related pathways, mainly involving mitochondrial outer membrane permeability, such as HIP1R and NMT1 (Barbu et al., 2020). Concurrently, genes like GAK (Nalls et al., 2014; Ma et al., 2015; Miyazaki et al., 2021) and PLEKHM1 (Barbu et al., 2020; Xu et al., 2020) have reported associations with PD via the lysosomal functional pathway.

Fisher’s exact test was conducted on 159 PD-related risk genes to ascertain more reliable genes associated with PD. As a result, 29 genes were identified from samples across 13 brain regions. In the progression of PD pathology, the loss of dopaminergic neurons in the substantia nigra can lead to oxidative stress (Trist et al., 2019), while α-Synuclein may trigger mitochondrial dysfunction (Rocha et al., 2018). Both these characteristics contribute to the neurodegenerative cascade reaction of PD. Additionally, ARSA, E2F1, SCNA, and other risk genes that are connected to the aforementioned two pathological characteristics have also been found. ARSA (Lee et al., 2019), acting as a molecular chaperone of SCNA (Dehay et al., 2015; Ferese et al., 2015; Du et al., 2020), plays a protective role in PD pathogenesis (Lee et al., 2019). DNA damage induces cell cycle reactivation and heightened E2F1 expression prompts neuronal apoptosis. Inhibiting cyclin activation, a potential drug target, demonstrates neuroprotective and anti-apoptotic effects in experimental models, suggesting the potential of the application of E2F1 in PD treatment (Verdaguer et al., 2007; Folch et al., 2012). Neuroinflammation is a significant player in PD pathology, with studies on numerous peripheral blood and cerebrospinal fluid samples from PD patients indicating that changes in immune function may exacerbate PD-related inflammation (Tansey et al., 2022). For instance, HLA-DRB1 (Le Guen et al., 2023) has been identified as an immune-related PD gene. Additionally, polymorphisms in STX1B (Wang et al., 2015) and CCDC62 (Lauterbach, 2012; Yi et al., 2023) are connected to PD. Numerous other DNA methylations, such as CRHR1 (Cheng et al., 2020; Rasmi et al., 2023) and ABCB9 (Chuang et al., 2017) have implications on PD development. CRHR1 signaling regulates embryonic neural stem cells, affecting brain development (Kwon et al., 2023). Furthermore, CRHR1 is involved in modulating glutamatergic and dopaminergic circuits, impacting neurotransmitter transmission and dopamine (Refojo et al., 2011), all of which contribute to PD development. Out of the 29 significant genes identified, 18 are involved in PD pathogenesis through multiple mechanisms.

We have discovered new risk genes for PD (Table 2), but their specific functions in PD remain unclear. Our exploration of significant genes and enrichment pathways can provide insights for future research on these genes’ roles in PD. These pathways, encompassing neuronal projection histogenesis, vesicle formation and transport, mitochondrial outer membrane permeability control, insulin secretion regulation, and Golgi tissue and functional pathways, have connections to PD pathology or have been previously described. Future PD research should therefore pay particular attention to these pathways. For instance, previous studies have shown that inhibition of CDC42 reduces various microglial activation properties, including increased cell body size, number of filopodia, and size of the Golgi apparatus. This reduction ultimately leads to a decrease in the unnecessary elimination of dopamine neurons. Therefore, CDC42 inhibitors hold promise as a potential alternative for the treatment of PD (Surviladze et al., 2010; Barcia et al., 2012; Ying et al., 2022). These findings provide valuable clues for in-depth research into their association and role in PD, indicating the need for further comprehensive investigation into PD risk factors.

Despite the numerous PD-associated targets unearthed through GWAS, high-throughput sequencing, molecular epidemiology, and other methodologies in recent decades, our understanding remains merely a fraction of the comprehensive knowledge necessary for diagnosing and treating PD. Leveraging the advanced TWAS approach, MR-JTI, a total of 159 genes linked to PD were identified. Fisher’s exact test was employed to validate more reliable PD risk genes. The discovery of these genes not only reaffirms previously documented PD-associated genes but also presents novel potential PD biomarkers warranting further investigation. Furthermore, future researchers could even analyze the 159 genes obtained in this study by integrating data from other omics, such as proteomics or epigenomics. By integrating data from different omics levels, genes involved in PD development through alternative mechanisms (post-translational modifications or epigenetic levels) could be identified, thereby gaining a more comprehensive understanding of the pathogenesis of PD.

## Data availability statement

The original contributions presented in the study are included in the article/Supplementary material, further inquiries can be directed to the corresponding author.

## Author contributions

Y-SW: Conceptualization, Data curation, Investigation, Software, Writing – original draft. W-HZ: Conceptualization, Investigation, Resources, Writing – original draft. T-HL: Conceptualization, Formal analysis, Funding acquisition, Software, Writing – review & editing. YS: Data curation, Formal analysis, Investigation, Writing – review & editing. Y-TX: Data curation, Formal analysis, Software, Writing – original draft. L-ZS: Writing – original draft. Q-YC: Writing – original draft. YT: Conceptualization, Data curation, Formal analysis, Funding acquisition, Investigation, Validation, Visualization, Writing – original draft, Writing – review & editing.

## Footnotes

^1^https://www.ebi.ac.uk/gwas/

^2^https://www.gtexportal.org/home/

^3^https://ctg.cncr.nl/software/MAGMA/ref_data/

^4^http://www.ncbi.nlm.nih.gov/geo

^5^https://david.ncifcrf.gov/tools.jsp

^6^https://www.genome.jp/kegg/
