# Supplementary material for: Joint-tissue integrative analysis identifies high-risk genes for Parkinson’s disease
Source: Front Neurosci. 2024 Mar 21;18:1309684. doi: 10.3389/fnins.2024.1309684 (PMC10991821; doi:10.3389/fnins.2024.1309684)
Supplement: Supplementary file 1 [file Data_Sheet_1.docx]

**Utilizing joint-tissue imputation to identify highly reliable risk loci of Parkinson’s disease**

**
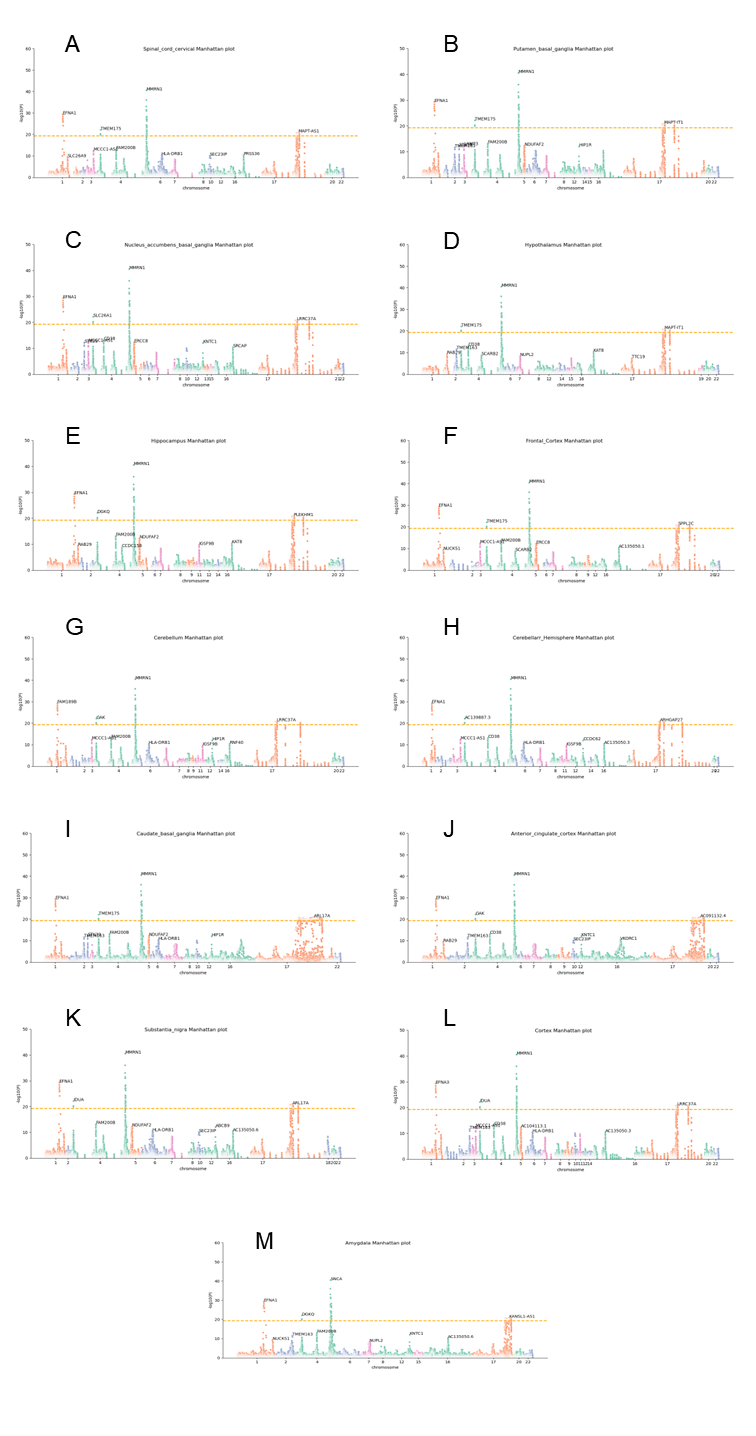
**

**Supplementary Figure 1. Manhattan maps obtained from MR JTI results of different brain regions**
